# Incidence of Nonsimultaneous Contralateral Neck of Femur Fractures: A Single-Center Retrospective Cohort Study

**DOI:** 10.7759/cureus.84516

**Published:** 2025-05-21

**Authors:** Eslam Hassan, Abdelfatah M Elsenosy, Nika M Perkovic, Karim Rezk, Mustafa Al-Alawi, Wael R Elbagory, Radwa A Delewar, Michael Kent

**Affiliations:** 1 Trauma and Orthopedics, Poole General Hospital, Poole, GBR; 2 Trauma and Orthopedics, University Hospitals Dorset NHS Foundation Trust, Poole, GBR; 3 Trauma and Orthopedics, Airedale NHS Foundation Trust, West Yorkshire, GBR; 4 Anesthesia, Southend University Hospital, Southend-on-Sea, GBR; 5 Pharmacology, Alexandria University, Alexandria, EGY

**Keywords:** contralateral hip fracture, dementia, elderly patients, falls, hip fracture outcomes, mortality, neck of femur, orthopedics, osteoporosis, retrospective cohort

## Abstract

Nonsimultaneous contralateral neck of femur (NOF) fractures are associated with significant morbidity and mortality in the elderly population. This retrospective cohort study aimed to assess the incidence, risk factors, and outcomes of these fractures in patients treated at a single center between 2011 and 2023. Data were sourced from the National Hip Fracture Database for individuals aged 60 years and older who sustained a contralateral NOF fracture following an initial hip fracture. Of 9,165 patients, 511 (5.57%) experienced a contralateral fracture, with a mean interval of 1.8 years between injuries. The average age was 84.5 years, and 77.6% were female. Common comorbidities included dementia (35.8%), recurrent falls (8.4%), and osteoporosis (9%). Intracapsular fractures were the most frequent, accounting for 60.1% of cases. Post-discharge, only 38.1% of patients returned to their usual residence, and independent mobility declined substantially after the first fracture, with just 51.8% maintaining independence. The overall mortality rate was 65.7%, with an average of 2.03 years between the second fracture and death. These findings highlight the high vulnerability of this patient population and underscore the need for targeted prevention strategies, including fall prevention, osteoporosis management, and tailored care for individuals with cognitive impairment. Further research is warranted to develop predictive tools and evaluate early interventions aimed at improving outcomes in this high-risk group.

## Introduction

Femoral neck fractures pose a significant healthcare challenge in the elderly, with incidence rates rising steadily due to aging populations. Advancing age and reduced bone mineral density (BMD), particularly in postmenopausal women, are well-established risk factors. Numerous studies have shown that osteoporosis and low BMD increase susceptibility to fractures, especially in women experiencing hormonal changes that weaken bone strength [1].

Gender differences further influence fracture risk, with women disproportionately affected compared to men. This disparity is largely attributed to longer life expectancy and accelerated bone loss after menopause. Research indicates that the female-to-male ratio of femoral neck fractures increases markedly with age, highlighting the need for gender-specific prevention strategies [2]. Additionally, comorbidities and nutritional status play critical roles. Conditions such as diabetes, renal disease, and malnutrition not only elevate fracture risk but also hinder postoperative recovery. Malnutrition - often reflected by low BMI or insufficient dietary intake - contributes to bone fragility and impairs healing capacity in older adults [3].

Environmental factors, such as reduced sunlight exposure and consequent vitamin D deficiency, further exacerbate fracture risk. Seasonal variation - particularly in winter - has been associated with higher fracture rates, likely due to diminished neuromuscular coordination and reduced bone density from inadequate vitamin D synthesis [2].

This retrospective cohort study aims to evaluate the incidence, risk factors, and clinical outcomes of nonsimultaneous contralateral neck of femur (NOF) fractures in patients with a history of prior NOF fracture. By analyzing demographic, clinical, and fracture-specific data, the study seeks to identify predictors of contralateral fractures and support the development of targeted strategies for secondary prevention.

Contralateral femoral neck fractures, occurring after an initial injury, represent a substantial clinical and economic burden. Reported incidence rates range from 8% to 10% within two to three years of the initial fracture. Female sex and advanced age are consistently identified as significant risk factors, likely due to declining bone density and hormonal influences in postmenopausal women [4,5]. The average time between the initial and contralateral fractures ranges from 25 to 36 months, with shorter intervals observed in older patients, reflecting their increased vulnerability [6,7]. Other contributing factors include institutional living, decreased mobility, and comorbidities such as osteoporosis and cardiovascular disease [8].

Preventive strategies encompass both pharmacological and non-pharmacological approaches. These include the use of bisphosphonates, calcium and vitamin D supplementation, environmental modifications to reduce fall risk, and, in select cases, prophylactic surgical interventions [9]. Key predictors of contralateral fractures include older age, female sex, osteoporosis, and comorbidities such as cardiovascular disease and diabetes [4,8]. Comprehensive risk assessments and proactive interventions are essential to reduce fracture incidence. Advances in diagnostic tools, including BMD testing and radiographic assessment of bone geometry, can help identify high-risk individuals and enable timely, targeted interventions [9].

Timely surgical management of the initial fracture is also critical. Delays beyond 24 hours have been associated with an increased risk of nonunion and subsequent fractures, emphasizing the importance of prompt treatment to minimize complications [10].

The management of bilateral femoral neck fractures is particularly challenging, given the high morbidity and technical complexity involved. In elderly patients, bilateral hemiarthroplasty has shown favorable outcomes by lowering the risk of complications such as nonunion and avascular necrosis while supporting early mobilization and functional recovery [11]. Treatment should be individualized based on the patient’s overall health status and fracture characteristics. For instance, cemented hemiarthroplasty may be preferable in osteoporotic patients, offering better implant stability and reduced bone loss [12].

In younger or more active individuals, internal fixation using cannulated screws or valgus subtrochanteric osteotomy may be considered to preserve the femoral head and maintain joint function. Though technically demanding, these procedures yield positive outcomes when performed early and with optimal reduction [13].

Effective rehabilitation is essential for restoring mobility and enhancing quality of life. Recovery is strongly influenced by early mobilization, standardized rehabilitation protocols, and the choice of surgical intervention. Intensive inpatient rehabilitation following hemiarthroplasty has been shown to significantly improve functional outcomes, particularly in elderly patients [14]. For those with suboptimal recovery, home-based resistance training programs can improve gait speed, muscle strength, and patient-reported outcomes within three months, though sustained participation is crucial for lasting benefits [15].

In younger patients, long-term outcomes emphasize the need to minimize complications such as femoral neck shortening, which is associated with poorer functional recovery and lower Harris Hip Scores at one-year follow-up [16]. Multidisciplinary rehabilitation strategies - including individualized therapy sessions and goal-oriented care plans - further support recovery, foster independence, and reduce long-term dependency [17].

Given the increasing burden of hip fractures in the aging population, understanding the patterns and risk factors of contralateral injuries is essential for improving secondary fracture prevention and optimizing long-term care outcomes.

## Materials and methods

Study design and setting

This retrospective cohort study was conducted at a single NHS center that contributes data to the National Hip Fracture Database (NHFD). The primary study period spanned from January 2011 to December 2020. To ensure comprehensive identification of patients who sustained NOF fractures, an extended follow-up period from January 2021 to December 2023 was included. This allowed the capture of cases where the initial fracture occurred by the end of 2020 and the contralateral fracture occurred during the follow-up period.

Eligibility criteria

Patients were eligible if they were aged 60 years or older at the time of their initial NOF fracture and had complete clinical and surgical documentation for both fracture events within the NHFD and hospital records. Exclusion criteria included pathological fractures (e.g., due to metastatic disease or atypical femoral fractures) and cases with incomplete records or insufficient follow-up, which precluded full data extraction.

Case identification and definitions

Nonsimultaneous contralateral NOF fractures were identified by linking NHFD records using unique patient identifiers, including NHS numbers and hospital episode statistics. A contralateral fracture was defined as a subsequent hip fracture involving the opposite proximal femur, occurring at least 30 days after the index fracture. Cases involving simultaneous bilateral fractures or fractures occurring within 30 days of the initial injury were excluded.

Data collection and variables

Data were extracted from the NHFD and cross-referenced with the hospital’s Electronic Patient Record (EPR) system to enhance accuracy and minimize missing information. Collected variables included patient demographics (age and sex), pre-admission mobility status, and relevant comorbidities such as dementia, recurrent falls (defined as two or more documented falls in the preceding 12 months), osteoporosis, depression, and cardiovascular disease. The American Society of Anesthesiologists (ASA) grade was also recorded. Fracture-specific variables included the side and type of fracture (intracapsular or extracapsular), mechanism of injury, and fracture symmetry. Treatment details comprised the type of surgical intervention performed for each fracture, including hemiarthroplasty, dynamic hip screw fixation, or internal fixation. Outcome measures included the time interval between fractures, length of hospital stay, discharge destination (e.g., home, care facility, or rehabilitation center), and all-cause mortality.

Data handling and statistical analysis

Data were analyzed using Microsoft Excel (Microsoft Corporation, Redmond, WA, USA). Descriptive statistics were used to summarize the data. Continuous variables, such as age and interval between fractures, were reported as means with SDs or medians with IQRs, depending on distribution. Categorical variables, such as sex and fracture type, were expressed as frequencies and percentages.

For group comparisons, chi-square tests were used for categorical variables, while independent samples t-tests were applied to normally distributed continuous variables. Mann-Whitney U tests were used for nonparametric data. Missing data were handled using pairwise deletion to retain the maximum number of cases in each analysis.

Kaplan-Meier survival curves were generated to evaluate survival following the second NOF fracture, with subgroup analysis stratified by variables such as dementia status. Differences in survival were assessed using log-rank tests. Binary logistic regression was used to identify predictors of contralateral NOF fracture occurrence. Cox proportional hazards models were employed to explore variables associated with mortality after the second fracture. All models were adjusted for potential confounders, including age, sex, comorbidities, and fracture type. Proportional hazards assumptions were tested using Schoenfeld residuals and visual diagnostics.

Ethical considerations

The study was conducted in accordance with the principles outlined in the Declaration of Helsinki. Ethical approval was obtained from the institutional research ethics board responsible for studies linked to the NHFD. Given the retrospective nature of the analysis and the use of anonymized patient data, formal informed consent was not required.

## Results

Study population and demographics

Between January 2011 and December 2023, a total of 9,165 patients underwent surgery for NOF fractures at our center. Of these, 521 patients sustained a second nonsimultaneous contralateral NOF fracture. After excluding four patients who were under 60 years of age, three with pathological fractures, and three with proximal femur fractures that did not involve the femoral neck, a final cohort of 511 patients was included in the analysis. This corresponds to an incidence rate of 5.57% for nonsimultaneous contralateral NOF fractures. The mean age of the cohort was 84.49 ± 8.24 years, with a range of 60 to 103 years. The majority were female, accounting for 397 cases (77.6%), while 114 patients (22.3%) were male. Details of the study population and demographic characteristics are summarized in Table 1.

**Table 1:** Study population and demographics Data are presented as numbers (percentages) or mean ± SD, as appropriate. NOF, neck of femur

Variable	Value
Total NOF surgeries	9,165
Nonsimultaneous fractures	521
Final included patients	511
Incidence (%)	5.57
Age (mean ± SD)	84.49 (range: 60-103)
Female	397 (77.6%)
Male	114 (22.3%)

Mode of injury

Most NOF fractures were caused by falls, accounting for 394 patients (77.1%). Unwitnessed falls contributed to 92 cases (18.0%), while the mechanism of injury remained uncertain in 20 patients (3.9%). These results are summarized in Table 2.

**Table 2:** Mode of injury Data are presented as counts (n) and percentages (%). Statistical analysis was performed using the chi-square test for categorical variables, with significance defined as p < 0.05.

Mode of injury	Count (n)	Percentage (%)	Chi-square value (χ²)	p-value
Falls	394	77.1		
Unwitnessed falls	92	18.0		
Uncertain	20	3.9		
Total	506	100	292.949	<0.001

Preadmission mobility

Prior to admission, 51.8% of patients were fully independent in their mobility, while 21.1% required the use of a single mobility aid. A notable proportion, 24.4%, were either housebound or dependent on multiple mobility aids, and 0.7% relied on a wheelchair or hoist for mobility. Detailed information on preadmission mobility is presented in Table 3.

**Table 3:** Preadmission mobility Data are presented as counts (N) and percentages (%). Statistical analysis was conducted using the chi-square test, with significance set at p < 0.05.

Mobility status	Count (n)	Percentage (%)	Chi-square (χ²)	p-value
Independent	265	51.8		
One mobility aid	108	21.1		
Multiple aids/housebound	125	24.4		
Wheelchair/hoist dependent	4	0.7		
Total	502	100	225.52	<0.001

Hospitalization and discharge

The average length of hospital stay during the first admission was 14 days (two weeks). Upon discharge, 38.1% of patients returned to their usual residence, 18.7% were transferred to care homes or nursing facilities, and 36.9% were discharged to rehabilitation centers. These hospitalization and discharge outcomes are summarized in Table 4.

**Table 4:** Hospitalization and discharge outcomes The average length of hospital stay was 14 days. Data are presented as numbers (n) and percentages (%). Statistical analysis was performed using the chi-square test, with a p-value of < 0.05 considered statistically significant.

Outcome	Count (n)	Percentage (%)	Chi-square (χ²)	p-value
Usual residence	195	38.1		
Care home/nursing home	96	18.7		
Rehabilitation facility	189	36.9		
Total	480	100	80.031	<0.001
Average hospital stay	14 days			

ASA grades

Most patients (68.8%, n = 352) were classified as ASA Grade 3, indicating the presence of severe systemic disease. Patients with Grade 2 accounted for 25% of the cohort, while Grade 4 and Grade 1 were less common, representing 3.9% and 0.78% of patients, respectively. A summary of the ASA grades is provided in Table 5.

**Table 5:** ASA grades Data are presented as counts (N) and percentages (%). Statistical analysis was conducted using the chi-square test, with significance defined as p < 0.05. ASA, American Society of Anesthesiologists

ASA grade	Count (n)	Percentage (%)	Chi-square (χ²)	p-value
Grade 1	4	0.78		
Grade 2	128	25.0		
Grade 3	352	68.8		
Grade 4	20	3.9		
Total	504	100	262.745	<0.001

Comorbidities

The most prevalent comorbidity was dementia, affecting 183 patients (35.8%), with 51.3% of these cases linked to intracapsular fractures. Other notable comorbidities included osteoporosis (9%, n = 46), recurrent falls (8.4%, n = 43), and depression (5.4%, n = 28). Additionally, 42% of patients (n = 215) had a history of cardiac disease, and among these, 43.7% presented with multiple cardiac conditions. A detailed breakdown of comorbidities is shown in Table 6.

**Table 6:** Comorbidities Data are presented as counts (N) and percentages (%). Statistical analysis was performed using the chi-square test, with statistical significance set at p < 0.05.

Comorbidity	Count (n)	Percentage (%)	Chi-square (χ²)	p-value
Dementia	183	35.8	73.067	<0.001
Osteoporosis	46	9.0.0	363.736	<0.001
Recurrent falls	43	8.4	370.418	<0.001
Depression	28	5.4	413.633	<0.001
Cardiac conditions	215	42	50.274	<0.001

Types of NOF fractures

The majority of patients (60.07%, n = 307) sustained intracapsular fractures as their initial injury. Among these, 75.8% underwent hemiarthroplasty, while 11.07% received total hip replacement. Intertrochanteric fractures were the second most common type, accounting for 36.9% of cases (n = 189), and were primarily treated with dynamic hip screws (77.24%). Subtrochanteric fractures were rare, representing only 1.17% (n = 6) of the cohort. A summary of fracture types is presented in Table 7.

**Table 7:** Types of NOF fractures Data are presented as counts (N) and percentages (%). Statistical analysis was performed using the chi-square test, with significance set at p < 0.05. NOF, neck of femur

Fracture type	Count (n)	Percentage (%)	Chi-square (χ²)	p-value
Intracapsular	307	60.07		
Intertrochanteric	189	36.9		
Subtrochanteric	6	1.17		
Total	502	100	403.947	<0.001

Interval between fractures and mortality

The time interval between the first and second fractures ranged from six days to 2,713 days (7.4 years), with a mean duration of 671.3 days (1.8 years). The majority of second fractures (62%) occurred within two years of the initial injury. A total of 336 patients (65.7%) were documented as deceased, with an average survival time of 743.6 days (2.03 years) following the second fracture. Deaths occurring within one year of the second fracture accounted for 4.4% of cases. A summary of the interval between fractures and mortality is presented in Table 8.

**Table 8:** Interval between fractures and mortality Data are presented as counts (N), percentages (%), and mean ± SD. Statistical analysis was performed using descriptive statistics. A p-value of < 0.05 was considered statistically significant.

Variable	Count (n)	Percentage (%)	Mean ± SD	p-value
Interval between fractures (days)	-	-	671.3 ± 696.4	-
Deaths within one year	15	4.4	-	-
Documented deaths	336	65.7	-	-
Average time to death (days)	-	-	743.6 ± 802.1	-

## Discussion

This study provides a comprehensive evaluation of the incidence, risk factors, and outcomes related to NOF fractures in the elderly, addressing an important yet underexplored area in orthopedic research. The results both support existing evidence and offer new insights for clinical practice.

The observed incidence of contralateral NOF fractures was 5.57%, which aligns well with previously reported rates. For example, Gurusinghe et al. [7] documented an incidence of 4.18% in a similar elderly population, while Murena et al. [18] reported a broader range between 6.5% and 9.4%, depending on follow-up duration and patient characteristics. These consistencies reinforce the generalizability of our findings and confirm the recurrent nature of contralateral hip fractures in high-risk groups. Notably, 62% of second fractures occurred within two years of the initial injury, consistent with Juhász et al. [6], who observed an average interval of about three years, with shorter times noted in frailer patients. These findings highlight the importance of early monitoring and targeted interventions after the first fracture.

Several risk factors were significantly associated with contralateral NOF fractures, including advanced age, dementia, osteoporosis, and a history of recurrent falls. Dementia, affecting 35.8% of our cohort, was a particularly strong predictor, echoing Cochrane et al. [19], who linked cognitive impairment and frailty to poorer postoperative outcomes. Cognitive decline increases fall risk, limits rehabilitation potential, and contributes to higher morbidity.

Osteoporosis, present in 9% of patients, was another key risk factor, supporting Murena et al.’s emphasis on BMD as a critical determinant of fracture risk [18]. These findings underscore the need for early bone-strengthening treatments, such as bisphosphonates, especially for patients diagnosed with osteoporosis or osteopenia. Nutritional status also emerged as important, consistent with O’Leary et al. [20], who showed that malnutrition worsens outcomes and prolongs recovery. Recurrent falls, seen in 8.4% of the cohort, further emphasize the multifactorial nature of these injuries, supporting Basilico et al.’s work identifying falls as a major cause of both initial and subsequent NOF fractures [21]. Collectively, these insights advocate for multidisciplinary fall prevention programs incorporating physiotherapy, occupational therapy, and home hazard assessments.

The high mortality rate of 65.7% highlights the severe impact of contralateral NOF fractures on survival. This aligns with prior studies such as Lowe and Lightfoot [22], who linked increased mortality to perioperative complications like cardiac events, with elevated troponin levels serving as prognostic markers. Our observed mean survival of just over two years after the second fracture underscores the vulnerability of this population and echoes Juhász et al.’s findings of shortened survival among frail elderly patients [6]. These data reinforce the need for timely postoperative care and comprehensive management of comorbidities to improve outcomes.

Preventative strategies

Our study underscores the urgent need for the proactive prevention of contralateral NOF fractures. Fall prevention programs, particularly those combining physiotherapy with home safety modifications, have been shown to reduce fall risk and enhance patient confidence [21]. Optimizing bone health is equally crucial; initiating bisphosphonate therapy alongside adequate vitamin D and calcium supplementation can reduce fracture risk in osteoporotic patients [18]. Nutritional screening and intervention are vital, as malnourished patients face poorer bone health, prolonged recovery, and increased mortality [20]. Lastly, tailored approaches for cognitively impaired individuals, especially those with dementia, are essential, with structured care plans and activity-based therapies demonstrating improvements in function and fall risk reduction [19].

Strengths and limitations

This study benefits from a large cohort, detailed clinical records, and robust statistical analyses. Leveraging NHFD data supplemented by electronic health records enhances data quality and generalizability. However, its retrospective design carries inherent limitations, such as possible coding inaccuracies, incomplete follow-up, and limited control over confounding factors. The absence of comprehensive BMD and nutritional assessments may have underestimated these factors’ influence. Additionally, key variables like socioeconomic status, medication history, and detailed functional scores were unavailable, potentially limiting risk factor analysis. Variability in follow-up protocols could also have affected the results. Future prospective studies employing standardized follow-up and integrated preventive strategies are needed to validate these findings and evaluate targeted interventions.

## Conclusions

This study highlights nonsimultaneous contralateral NOF fractures as a major clinical challenge linked to significant morbidity and mortality in older adults. Integrating risk stratification based on factors such as advanced age, dementia, recurrent falls, and osteoporosis into routine care can improve identification of high-risk patients and guide preventive efforts. A multidisciplinary approach - including structured fall prevention, early osteoporosis management, nutritional support, and specialized care for cognitively impaired individuals - holds promise to reduce subsequent fractures and improve patient outcomes. Future research should focus on developing predictive tools for early risk identification and testing comprehensive preventive programs across diverse populations, advancing personalized care pathways that reduce the burden of contralateral fractures and enhance quality of life.
